# An extreme value analysis of daily new cases of COVID-19 in Africa

**DOI:** 10.3389/fpubh.2025.1546404

**Published:** 2025-01-31

**Authors:** Saralees Nadarajah, Adamu Abubakar Umar

**Affiliations:** ^1^Department of Mathematics, University of Manchester, Manchester, United Kingdom; ^2^Department of Statistics, Ahmadu Bello University, Zaria, Nigeria

**Keywords:** generalized extreme value distribution, Kolmogorov-Smirnov test, linear trend, quadratic trend, estimation

## Abstract

Modeling COVID-19 cases in Africa is crucial for developing effective public health strategies, allocating resources efficiently, and mitigating the impact of the pandemic on vulnerable populations. A recent paper by the first author provided an extreme value analysis of daily new cases of COVID-19 from sixteen countries in west Africa. In this paper, we broaden our analysis to encompass data spanning all fifty four African nations over a period of forty four months. We identified extreme values as the monthly maximums of daily new cases. Utilizing the generalized extreme value distribution, we fitted the data, allowing two of its three parameters to vary linearly or quadratically in relation to the month number. Twenty six countries demonstrated significant downward trends in monthly maximums. Two countries demonstrated significant upward trends in monthly maximums. Nineteen countries demonstrated significant quadratic trends where monthly maximums initially increased before decreasing. The sharpest and weakest of the downward trends with respect to location were for Mali and Liberia, respectively. The sharpest and weakest of the downward trends with respect to scale were for Egypt and Libya, respectively. Recommendations are given for each country. We evaluated the adequacy of fits through probability plots and the Kolmogorov-Smirnov test. Subsequently, the fitted models were employed to determine quantiles of the monthly maximum of new cases, as well as their limits extrapolated to infinite month numbers.

## 1 Introduction

Numerous papers have been published on the daily new cases and deaths during the COVID-19 pandemic; see, for example, (1–3). However, the focus often lies on extreme values associated with these variables. There have been relatively few papers dedicated to modeling extreme values of COVID-19 cases or deaths. The ones we are aware of are: an exponential smoothing Holt-Winters based-approach to model extreme values of COVID-19 cases in central Java (4); use of extreme values of COVID-19 deaths in Japan, Saudi Arabia and Romania to illustrate the fit of a proposed distribution (5); fit of the Gumbel extreme value distribution to the maximum of daily COVID-19 cases in Argentina, Brazil, China, Colombia, France, Germany, India, Indonesia, Iran, Italy, Mexico, Poland, Russia, Spain, the United Kingdom and the United States (6); modeling of the maximum number of weekly cases and deaths caused by the COVID-19 pandemic in Iraq (7).

However, there exist additional studies examining the effects of the pandemic on extreme values of various other variables. For example, the extreme tail behavior of NIFTY 50 index during the COVID-19 pandemic was investigated by (8). Effects of COVID-19 on extreme values of cryptocoin markets were forecasted by (9). The dependence between the extreme values of IDX Composite Indices, Straits Times Indices and Kuala Lumpur Stock Exchange Indices during the COVID-19 pandemic was modeled by (10). Pricing of extreme mortality risk in the wake of the COVID-19 pandemic was studied by (11).

Extreme value analysis of daily COVID-19 cases is crucial for Africa due to its unique public health challenges and resource limitations. Africa's healthcare systems often face capacity constraints, with limited hospital beds, ICU units, and healthcare personnel. By analyzing the extreme values–such as the highest spikes in daily cases – authorities can predict worst-case scenarios and prepare accordingly. This helps in resource allocation, ensuring that critical supplies like oxygen, ventilators, and medicines are available when demand peaks. It also informs policy decisions, such as the timing and intensity of lockdowns or social distancing measures, to prevent healthcare systems from being overwhelmed.

Moreover, extreme value analysis is essential in understanding the variability and potential future risks posed by COVID-19 in African countries. Many regions in Africa face data scarcity and underreporting, making traditional forecasting methods less reliable. Extreme value analysis focuses on the tail end of distributions, which is particularly useful in understanding extreme outbreaks that may disproportionately affect vulnerable populations. By identifying these outlier events and their probabilities, governments and organizations can design targeted interventions to mitigate the impact on socioeconomically disadvantaged communities, thereby reducing overall morbidity and mortality. This approach is critical for ensuring equitable and effective pandemic response strategies across the continent.

The application of extreme value analysis to daily new cases in West Africa was pioneered by (12), marking a significant advancement in the field. Their study focused on sixteen countries in West Africa, offering a comprehensive analysis of daily new cases. Utilizing a general extreme value distribution, they effectively accounted for trends within the data. Extreme values were defined as the monthly maximums of daily new cases, providing a robust foundation for their analysis.

No subsequent paper has been identified since (12) that offers an extreme value analysis of daily new cases in Africa. The objective of this study is to replicate the models introduced by (12) using data from all African countries. These fitted models serve to estimate quantiles of the monthly maximum of new cases and ascertain their limits as the month number approaches infinity.

The paper is structured as follows: Section 2 presents the model used to perform the extreme value analysis; Section 3 describes the data, the results obtained from fitting the generalized extreme model, along with their discussion; Section 4 outlines the conclusions drawn from the analysis and gives recommendations based on the conclusions and future work. All tables are given in the appendix. All computations in this paper were conducted using the R software (13).

## 2 Methods

Following arguments in (12), we can show that the distribution of the monthly maximums of daily new cases can be approximated by the generalized extreme value distribution specified by the cumulative distribution function,


(1) $$\exp\left[-{\left(1+\xi \frac{x-\mu }{\sigma }\right)}^{-\frac{1}{\xi }}\right],$$

for $\mu -\frac{\sigma }{\xi }\leq x<\infty$ if ξ > 0, −∞ < *x* < ∞ if ξ = 0 and $-\infty <x\leq \mu -\frac{\sigma }{\xi }$ if ξ < 0, where −∞ < μ < ∞ denotes a location parameter, σ > 0 denotes a scale parameter and −∞ < ξ < ∞ denotes a shape parameter.

The model in (Equation 1) is referred to as the generalized extreme value model. It was fitted to data from west Africa with its location and scale parameters allowed to vary linearly or quadratically with respect to month number counted from January 2020 (12). The following models were fitted: the constant model which is the same as (Equation 1); the linear location model with the location parameter allowed to vary linearly with respect to month number; the quadratic location model with the location parameter allowed to vary quadratically with respect to month number; the linear scale model with the scale parameter allowed to vary linearly with respect to month number; the quadratic scale model with the scale parameter allowed to vary quadratically with respect to month number; the linear location and linear scale model with both the location and scale parameters allowed to vary linearly with respect to month number; the quadratic location and linear scale model with the location parameter allowed to vary quadratically with respect to month number while the scale parameter allowed to vary linearly with respect to month number; the linear location and quadratic scale model with the location parameter allowed to vary linearly with respect to month number while the scale parameter allowed to vary quadratically with respect to month number; the quadratic location and quadratic scale model with both the location and scale parameters allowed to vary quadratically with respect to month number.

## 3 Results and discussion

The data sourced from https://ourworldindata.org/covid-cases comprises daily new cases in each African country spanning from 3 January 2020 to 31 May 2024. The data in https://ourworldindata.org/covid-cases is sourced from reliable institutions like national health agencies, the World Health Organization (WHO), and the European Centre for Disease Prevention and Control (ECDC). It includes daily updates on confirmed COVID-19 cases, deaths, vaccinations, testing, and hospitalizations across countries. The platform standardizes this data to allow cross-country comparisons and trends analysis. It provides visualizations such as charts and maps to facilitate understanding of the pandemic's trajectory globally and locally. The dataset is openly accessible, enabling researchers, policymakers, and the public to track the pandemic, evaluate policy impacts, and inform decision-making with evidence-based insights.

The data cover the countries include Botswana, Lesotho, Namibia, South Africa, Eswatini, Algeria, Egypt, Libya, Morocco, Sudan, Tunisia, Angola, Cameroon, the Central African Republic, Chad, the Democratic Republic of Congo, Congo, Equatorial Guinea, Gabon, Sao Tome, Benin, Burkina Faso, Cape Verde, Cote d'Ivoire, the Gambia, Ghana, Guinea, Guinea-Bissau, Liberia, Mali, Mauritania, Niger, Nigeria, Senegal, Sierra Leone, Togo, Burundi, Comoros, Djibouti, Eritrea, Ethiopia, Kenya, Madagascar, Malawi, Mauritius, Mozambique, Rwanda, Seychelles, Somalia, South Sudan, Tanzania, Uganda, Zambia, and Zimbabwe. We focus on monthly maximums of daily new cases, corresponding to month numbers 1 through 53. Table 1 in Appendix presents some summary statistics of this dataset.

All countries experience months without new cases. Morocco has the highest first quartile of monthly new cases, followed by the Democratic Republic of Congo, Zambia, Zimbabwe, Angola, Ethiopia, and several other countries. Remaining countries have a first quartile of zero. South Africa shows the highest median of monthly cases, followed by Tunisia, Morocco, Mauritius, Zambia, Egypt, and others. It also has the largest mean and third quartile, with Tunisia, Botswana, Morocco, and Libya trailing in both. Botswana reports the highest monthly peak, followed by South Africa, Tunisia, Uganda, and Tanzania. Standard deviation is highest in Botswana, South Africa, and Tunisia, indicating variation across countries. Skewness of new cases is consistently positive, highest in Uganda, followed by Mauritania and Liberia, while kurtosis consistently exceeds 3, indicating heavy-tailed data, with the highest values in Uganda, Mauritania, and Liberia.

Figures A8 to A13 in (14) depict scatter plots illustrating the monthly maximums data for the fifty four countries. Additionally, the figures include smoothed versions of the scatter plots using lowess (15, 16) technique. Many countries demonstrate noticeable trends with respect to month number, which seem to follow either linear or quadratic patterns.

We applied the generalized extreme value model to fit the monthly maximums of new cases. The estimation method assumes data independence, which we assessed using a runs test. The resulting *p*-values given in (14) show no evidence against independence for any of the countries.

We employed various models, including constant, linear location, quadratic location, linear scale, quadratic scale, linear location and linear scale, quadratic location and linear scale, linear location and quadratic scale, and quadratic location and quadratic scale, as outlined in Section 3 of (12). The selection of the best fitting models was based on several criteria, including the likelihood ratio test for comparing likelihood values (17), the Akaike information criterion (AIC) proposed by (18) and the Bayesian information criterion (BIC) introduced by (19). The best fitted models corresponded to the smallest AIC and the BIC values.

Table 2 in Appendix gives the parameter estimates, standard errors, log-likelihood values and values of Akaike information and Bayesian information criteria when the best fitting model was the constant model. Table 3 in Appendix gives the parameter estimates, standard errors, log-likelihood values and values of Akaike information and Bayesian information criteria when the best fitting model was the linear location model. Table 4 in Appendix gives the parameter estimates, standard errors, log-likelihood values and values of Akaike information and Bayesian information criteria when the best fitting model was the quadratic location model. Table 5 in Appendix gives the parameter estimates, standard errors, log-likelihood values and values of Akaike information and Bayesian information criteria when the best fitting model was the linear location and linear scale model. Table 6 in Appendix gives the parameter estimates, standard errors, log-likelihood values and values of Akaike information and Bayesian information criteria when the best fitting model was the quadratic location and linear scale model. Table 7 in Appendix gives the parameter estimates, standard errors, log-likelihood values and values of Akaike information and Bayesian information criteria when the best fitting model was the linear location and quadratic scale model. Table 8 in Appendix gives the parameter estimates, standard errors, log-likelihood values and values of Akaike information and Bayesian information criteria when the best fitting model was the quadratic location and quadratic scale model. The standard errors were obtained by the bootstrap procedure outlined in Section 3 in (12). We see that all of the standard errors are less than the parameter estimates in magnitude.

The constant model gave the best fit for the Central African Republic, Chad, Equitorial Guinea, Cape Verde, the Gambia, Guinea-Bissau and Djibouti. The linear location model gave the best fit for Sao Tome, Liberia, South Sudan and Uganda. The quadratic location model gave the best fit for South Africa, the Democratic Republic of Congo, Gabon, Tanzania and Zambia. The linear location and linear scale model gave the best fit for Eswatini, Morocco, Sudan, Burkina Faso, Ghana, Mali, Mauritania, Senegal, Sierra Leone, Togo, Madagascar and Malawi. The quadratic location and linear scale model gave the best fit for Egypt, Libya, Tunisia, Angola, Benin, Cote d'Ivoire, Guinea, Niger, Nigeria, Mauritius and Mozambique. The linear location and quadratic scale model gave the best fit for Comoros. The quadratic location and quadratic scale model gave the best fit for Botswana, Lesotho, Namibia, Algeria, Cameroon, Congo, Burundi, Eritrea, Ethiopia, Kenya, Rwanda, Seychelles, Somalia and Zimbabwe.

The probability plots of the best fitting models are shown in Figures A14 to A19 in (14). The plots show that best fitting models are reasonable except for Lesotho, Egypt, Tunisia, Congo and Gabon. The *p*-values of the Kolmogorov-Smirnov test (20, 21) obtained using the resampling method described in (12) are given in (14). They showed that the best fitting models provided adequate fits except for Lesotho, Egypt, Tunisia, Congo and Gabon.

**Figure 1:**
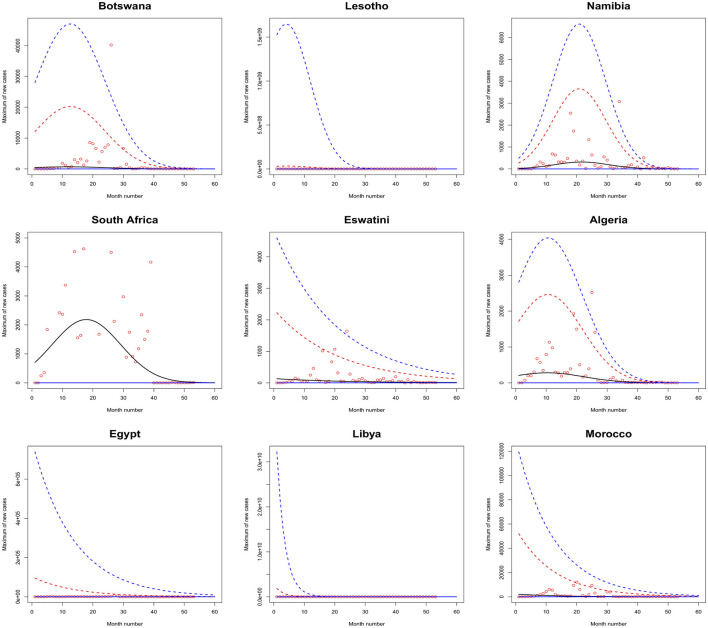
Fitted 2.5 (solid blue), 5 (solid red), 50 (solid black), 95 (broken red) and 97.5 (broken blue) percent quantile functions vs. month number counting from January of 2020 for Botswana, Lesotho, Namibia, South Africa, Eswatini, Algeria, Egypt, Libya, and Morocco.

**Figure 2:**
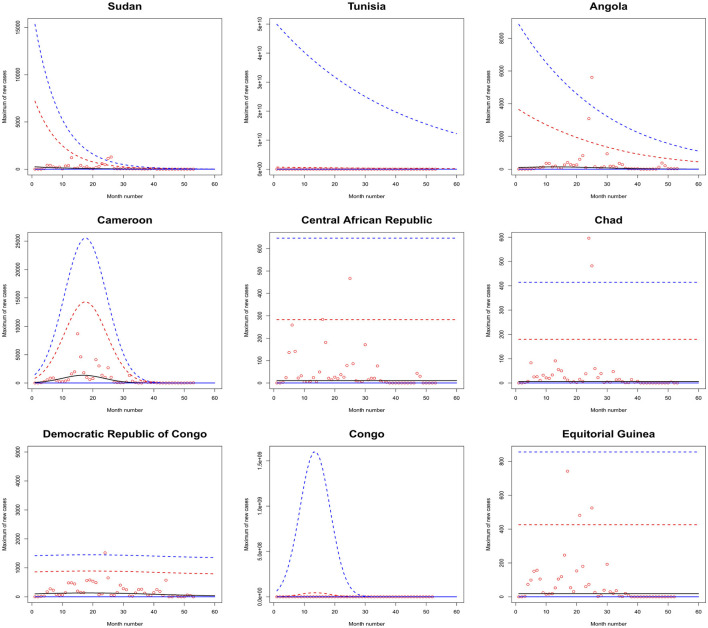
Fitted 2.5 (solid blue), 5 (solid red), 50 (solid black), 95 (broken red) and 97.5 (broken blue) percent quantile functions vs. month number counting from January of 2020 for Sudan, Tunisia, Angola, Cameroon, the Central African Republic, Chad, the Democratic Republic of Congo, Congo, and Equitorial Guinea.

**Figure 3:**
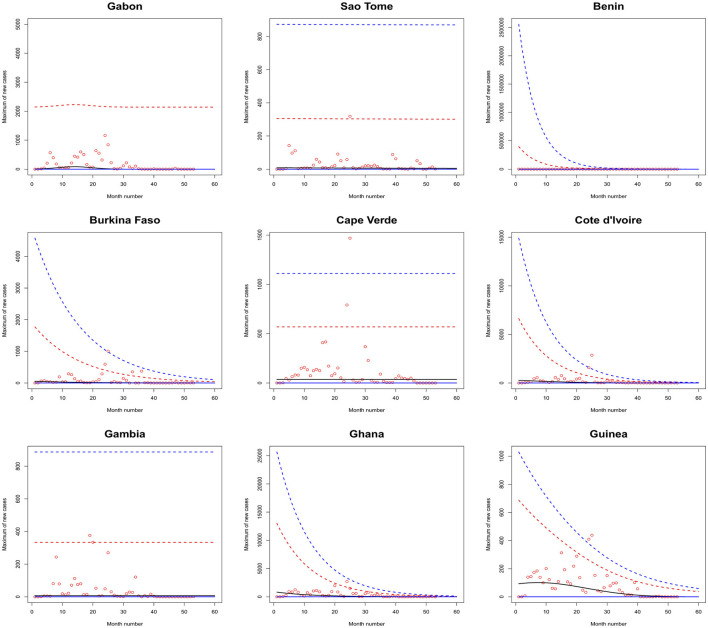
Fitted 2.5 (solid blue), 5 (solid red), 50 (solid black), 95 (broken red) and 97.5 (broken blue) percent quantile functions vs. month number counting from January of 2020 for Gabon, Sao Tome, Benin, Burkina Faso, Cape Verde, Cote d'Ivoire, the Gambia, Ghana, and Guinea.

**Figure 4:**
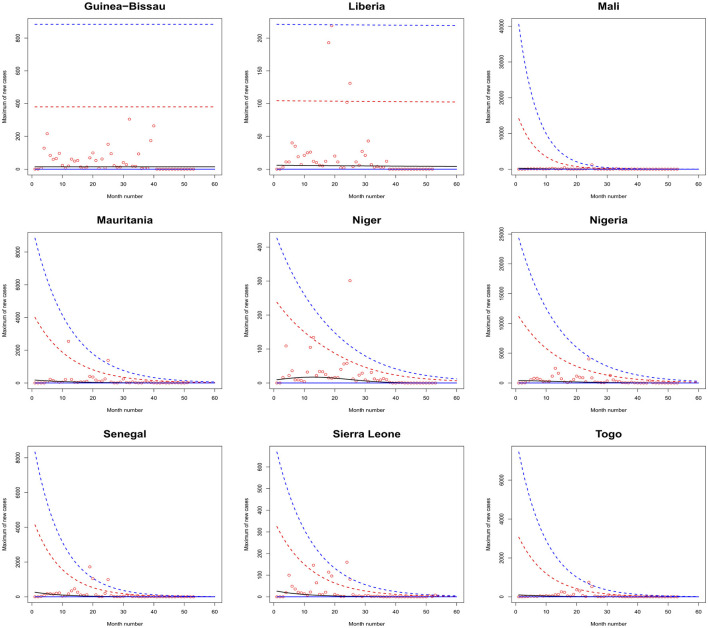
Fitted 2.5 (solid blue), 5 (solid red), 50 (solid black), 95 (broken red) and 97.5 (broken blue) percent quantile functions vs. month number counting from January of 2020 for Guinea-Bissau, Liberia, Mali, Mauritania, Niger, Nigeria, Senegal, Sierra Leone, and Togo.

**Figure 5:**
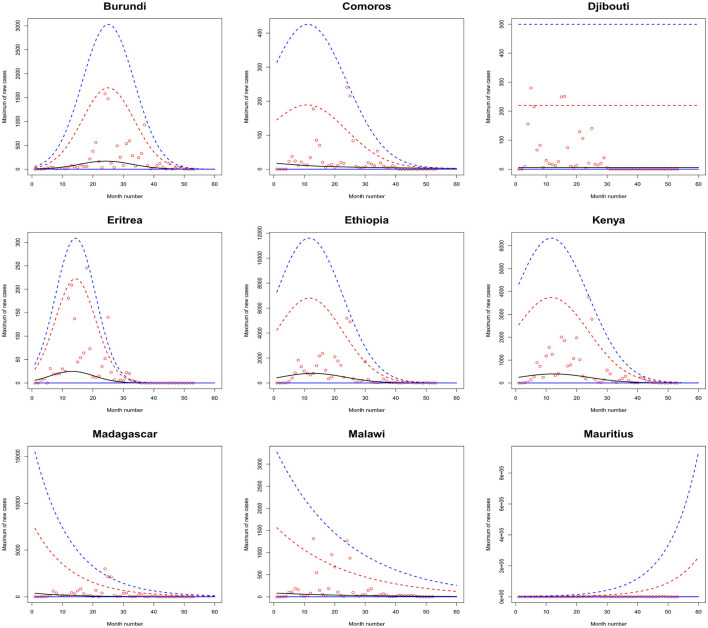
Fitted 2.5 (solid blue), 5 (solid red), 50 (solid black), 95 (broken red) and 97.5 (broken blue) percent quantile functions vs. month number counting from January of 2020 for Burundi, Comoros, Djibouti, Eritrea, Ethiopia, Kenya, Madagascar, Malawi, and Mauritius.

**Figure 6:**
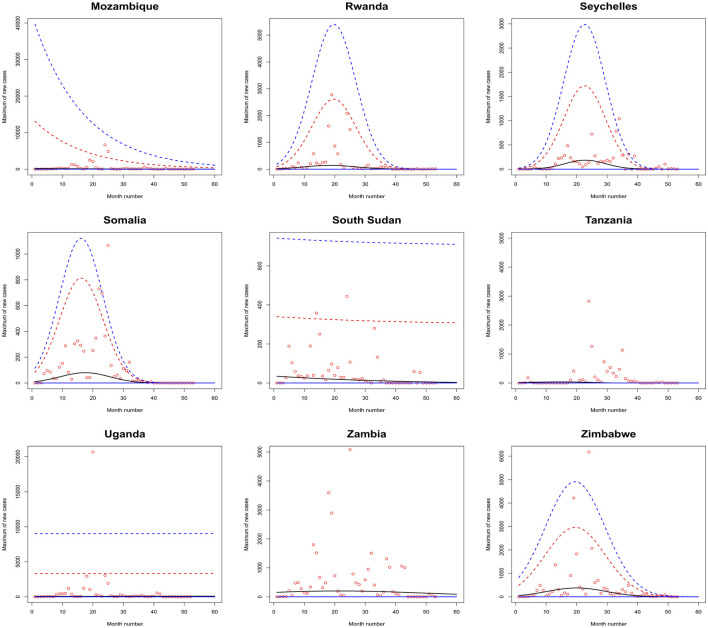
Fitted 2.5 (solid blue), 5 (solid red), 50 (solid black), 95 (broken red) and 97.5 (broken blue) percent quantile functions vs. month number counting from January of 2020 for Mozambique, Rwanda, Seychelles, Somalia, South Sudan, Tanzania, Uganda, Zambia, and Zimbabwe.

Figures 1–6 plot the 2.5, 5, 50, 95, and 97.5 percent quantile functions vs. the month number ranging from 1 to 60 for the best fitting models. The *q*th percent quantile function for *q* = 2.5, 5, 50, 95, 97.5 was computed by


(2) $$\hat{Q}(q)=\{\begin{array}{ll} 0, & \text{if }q\leq \hat{p},\\ \hat{\mu }+\frac{\hat{\sigma }}{\hat{a_{3}}}\left\{{\left[-\log\left(\frac{q-\hat{p}}{1-\hat{p}}\right)\right]}^{-\hat{a_{3}}}-1\right\}, & \text{if }q>\hat{p}, \end{array}$$

where $\hat{p}$ is the proportion of zeros in the data, $\hat{\mu }$ is one of


$$\hat{\mu }=\{\begin{array}{ll} \exp\left(\hat{a_{1}}\right), & \text{for constant model,}\\ \; & \;\\ \exp\left[\hat{a_{1}}+\hat{b_{1}}\cdot \text{month no}\right], & \text{for linear location model,}\\ \; & \text{linear location and linear scale model,}\\ \; & \text{linear location and quadratic scale model,}\\ \; & \;\\ \exp\left[\hat{a_{1}}+\hat{b_{1}}\cdot \text{month no}+\hat{c_{1}}\cdot {\text{month no}}^{2}\right], & \text{for quadratic location model,}\\ \; & \text{quadratic location and linear scale model,}\\ \; & \text{quadratic location and quadratic scale model} \end{array}$$

and $\hat{\sigma }$ is one of


$$\hat{\sigma }=\{\begin{array}{ll} \exp\left(\hat{a_{2}}\right), & \text{for constant model,}\\ \; & \text{for linear location model,}\\ \; & \text{for quadratic location model,}\\ \; & \;\\ \exp\left[\hat{a_{2}}+\hat{b_{2}}\cdot \text{month no}\right], & \text{linear location and linear scale model,}\\ \; & \text{quadratic location and linear scale model,}\\ \; & \;\\ \exp\left[\hat{a_{2}}+\hat{b_{2}}\cdot \text{month no}+\hat{c_{2}}\cdot {\text{month no}}^{2}\right], & \text{quadratic location and quadratic scale model,}\\ \; & \text{linear location and quadratic scale model.} \end{array}$$

The monthly maximum of new cases for Sao Tome, Liberia and South Sudan steadily decrease with respect to the month number. For these countries, the location parameter estimate decreases by a multiple of exp(−1.284), exp(−0.474) and exp(−2.471) for every unit increase in month number. The monthly maximum of new cases for Uganda steadily increases with respect to the month number. The corresponding location parameter estimate increases by a multiple of exp(0.515) for every unit increase in month number.

The monthly maximum of new cases for South Africa, the Democratic Republic of Congo, Gabon, Tanzania and Zambia initially increase and then decrease. The monthly maximum of new cases for these countries reach their highest values in June 2021, July 2021, February 2021, March 2021 and September 2021.

The monthly maximum of new cases for Eswatini, Morocco, Sudan, Burkina Faso, Ghana, Mali, Mauritania, Senegal, Sierra Leone, Togo, Madagascar and Malawi steadily decrease with respect to the month number. For these countries, the location parameter estimate decreases by a multiple of exp (−4.840), exp (−7.986), exp (−12.028), exp (−6.417), exp (−8.895), exp (−15.681), exp (−8.442), exp (−10.815), exp (−8.496), exp (−10.525), exp (−8.200) and exp (−4.334) for every unit increase in month number. The corresponding scale parameter estimate decreases by a multiple of exp(−4.261), exp (−7.551), exp (−8.254), exp (−6.653), exp (−8.511), exp (−12.539), exp (−7.837), exp (−10.534), exp (−9.636), exp (−8.883), exp (−7.732) and exp (−3.707) for every unit increase in month number.

The monthly maximum of new cases for Egypt, Libya, Tunisia, Angola, Benin, Cote d'Ivoire, Guinea, Niger, Nigeria and Mozambique generally decrease with respect to the month number. The corresponding scale parameter estimate decreases by a multiple of exp (−93.152), exp (−2.374), exp (−57.431), exp (−25.243), exp (−7.297), exp (−13.815), exp (−18.745), exp (−30.502), exp (−11.604) and exp (−7.105) for every unit increase in month number. The monthly maximum of new cases for Mauritius generally increases with respect to the month number. The corresponding scale parameter estimate decreases by a multiple of exp(−38.269) for every unit increase in month number.

The monthly maximum of new cases for Comoros generally decreases with respect to the month number. The corresponding location parameter estimate increases by a multiple of exp(7.173) for every unit increase in month number.

The monthly maximum of new cases for Botswana, Lesotho, Namibia, Algeria, Cameroon, Congo, Burundi, Eritrea, Ethiopia, Kenya, Rwanda, Seychelles, Somalia and Zimbabwe initially increase before decreasing with respect to the month number. The corresponding location parameter estimates reach their highest values in December 2020, April 2020, September 2021, October 2020, May 2021, July 2023, January 2022, March 2021, February 2021, February 2021, July 2021, December 2021, May 2021 and August 2021. The corresponding scale parameter estimates reach their highest values in February 2021, April 2020, August 2021, October 2020, May 2021, March 2021, February 2022, March 2021, January 2021, January 2021, August 2021, December 2021, April 2021 and July 2021.

Table 9 in Appendix gives the limit of (Equation 2) as the month number is taken to infinity. The 2.5 percent and 5 percent quantiles are equal to zero for all the countries. The 50 percent, 95 percent and 97.5 percent quartiles are infinite for Uganda and Congo.

Considering finite values, the 50 percent quartile is the largest Libya, followed by Mozambique, Cote d'Ivoire, Cape Verde, Angola, Nigeria, Zambia, Equitorial Guinea, Guinea-Bissau, the Democratic Republic of Congo, the Central African Republic, the Gambia, Chad, Djibouti, South Africa, Mauritius and Sao Tome. For the remaining countries the 50 percent quartile is zero.

Considering finite values, the 95 percent quartile is the largest Libya, followed by Tunisia, Benin, South Africa, Egypt, Mozambique, Tanzania, Nigeria, Cote d'Ivoire, Zambia, Angola, Gabon, the Democratic Republic of Congo, Guinea, Cape Verde, Mauritius, Equitorial Guinea, Guinea-Bissau, Gambia, South Sudan, Sao Tome, the Central African Republic, Niger, Djibouti, Chad and Liberia. For the remaining countries the 95 percent quartile is zero.

Considering finite values, the 97.5 percent quartile is the largest Tunisia, followed by Libya, Benin, South Africa, Egypt, Tanzania, Mozambique, Nigeria, Cote d'Ivoire, Zambia, Gabon, Angola, Mauritius, the Democratic Republic of Congo, Cape Verde, Guinea, Gambia, Guinea-Bissau, Sao Tome, Equitorial Guinea, South Sudan, the Central African Republic, Djibouti, Chad, Niger and Liberia. For the remaining countries the 97.5 percent quartile is zero.

The African continent comprises five regions, namely Northern Africa, Central Africa, Eastern Africa, Western Africa, and Southern Africa. Northern Africa has seven countries including Algeria, Egypt, Libya, Morocco, Tunisia, Sudan, and South Sudan. Central Africa has nine countries including Angola, Cameroon, Equatorial Guinea, Gabon, Congo, Chad, the Central African Republic, the Democratic Republic of Congo, and Sao Tome. Eastern Africa has seventeen countries including Burundi, Eritrea, Madagascar, Mauritius, Somalia, Comoros, Ethiopia, Rwanda, Djibouti, Kenya, Seychelles, Uganda, Mozambique, Zambia, Malawi, Tanzania, and Zimbabwe. Western Africa has sixteen countries including Benin, Liberia, Burkina Faso, Gambia, Mali, Ghana, Mauritania, Senegal, Cape Verde, Cote d'Ivoire, Guinea, Niger, Sierra Leone, Guinea-Bissau, Nigeria and Togo. Southern Africa has five countries including Lesotho, Eswatini, Botswana, Namibia, and South Africa.

Southern Africa reported the highest of the total number of maximum daily new cases (84,408), followed by Eastern Africa (83,238), Northern Africa (54,838) and Western Africa (23,539). Central Africa reported the lowest of the total number of maximum daily new cases (19,889).

Eastern Africa results show that monthly maximums of new cases increase with respect to month number for two countries while for others they initially increase and later decrease. Central Africa results indicate that monthly maximums of new cases decrease for two countries while for others they initially increase and then decrease. Southern Africa results show that monthly maximums of new cases decrease for one country while for others they initially increase and later decrease. Northern Africa results indicate that monthly maximums of new cases decrease for six countries while for the remaining country they initially increase and then decrease. Western Africa results show that monthly maximums of new cases steadily decrease for nine countries while for others they generally decrease.

In Northern Africa, nations like Morocco, Tunisia, and Egypt demonstrated decreasing trends due to robust healthcare systems, timely interventions, and effective immunization programs. In contrast, countries such as Algeria, Libya, and Sudan experienced initial surges followed by declines, influenced by political instability, inadequate infrastructure, and delayed responses. In Central Africa, countries like the Central African Republic and Chad, inadequate healthcare systems and governance challenges exacerbated the pandemic's impact, while more structured responses in Gabon and Equatorial Guinea led to improved outcomes. Similarly, initial surges in the Congo and Cameroon were driven by inadequate infrastructure, political instability, and limited access to vaccines and testing, though some regions have achieved notable progress with decreasing trends. In Eastern Africa, nations with strong governance and healthcare systems, like Kenya and Rwanda, managed the pandemic more effectively, while others, such as Somalia and Tanzania, faced significant challenges. Countries like Djibouti, Madagascar, Malawi, Mozambique, and Uganda saw declining trends due to timely interventions and improved healthcare, while Mauritius experienced increasing cases. Some countries experienced an initial surge followed by a decrease. In Western Africa, countries like Ghana and Senegal achieved better outcomes due to robust governance, timely interventions, and consistent immunization programs, while others, including Mali, Guinea, and Burkina Faso, struggled with political instability, inadequate healthcare facilities, and resource constraints. Overall, nations like Senegal, Ghana, Sierra Leone, Niger, Mauritania, Nigeria, Mali, and Togo exhibited a decreasing trend in cases, attributed to strengthened healthcare systems and proactive measures, although some initially experienced a surge before cases declined. In Southern Africa, nations like Botswana and South Africa demonstrated superior performance due to robust infrastructure and timely interventions, while others, such as Malawi and Lesotho, struggled with resource constraints and logistical challenges. The region exhibited initial increases in cases followed by declines in countries like Botswana, Namibia, Lesotho, and South Africa, whereas Eswatini maintained a decreasing trend, attributed to effective vaccination campaigns, advanced healthcare systems, and proactive public health measures.

In general, the complex combination of demographic, socioeconomic, healthcare, and geopolitical factors influence COVID-19 patterns in African regions. The African continent's youthful population and previous epidemic experience helped to minimize some of the results, while socioeconomic difficulties, vaccination shortcomings and healthcare system failures increased the pandemic's impact. The implications of COVID-19 results and trends in African regions are multifaceted, spanning public health, economics, education, and governance. Addressing these implications requires sustained investments in healthcare systems, education, and economic recovery, alongside strengthened governance, regional collaboration, and international partnerships.

For countries where COVID-19 cases are expected to increase, governments may consider a range of policy measures to manage the situation effectively. Strengthening public health measures like mask mandates, social distancing, and capacity limits in public spaces can help reduce transmission rates. Vaccination campaigns should be intensified by expanding vaccine access, addressing hesitancy through awareness efforts, and ensuring efficient distribution. Enhancing testing and contact tracing can promptly identify and isolate cases, while bolstering healthcare system preparedness, including increasing hospital capacity and supporting healthcare workers, ensures readiness for patient surges. Travel restrictions and quarantine measures may prevent the importation of new variants, while policies for schools and workplaces might include remote learning or telework and in-person safety protocols. Targeted interventions for high-risk populations or areas, clear and transparent communication to build public trust, and economic support for affected individuals and businesses are also critical. Finally, continuous monitoring of data and adaptive policymaking ensure responses remain effective as conditions evolve. A coordinated approach addressing public health, healthcare, economic, and social considerations is essential for mitigating the impact of rising cases.

For countries where COVID-19 cases are forecasted to decrease, it is vital to maintain vigilance and proactive measures to ensure sustained control of the pandemic and support long-term recovery. Governments should continue emphasizing widespread vaccination to achieve herd immunity and prevent future outbreaks while maintaining basic public health measures such as mask-wearing and hand hygiene to counter potential resurgences, especially with new variants. This period of lower cases provides an opportunity to strengthen healthcare infrastructure by investing in hospital capacity, medical supplies, and workforce training. Efforts should also shift toward economic recovery, supporting businesses, restoring jobs, and addressing pandemic-exacerbated socioeconomic disparities. Safe reopening of schools and workplaces requires hybrid models, safety protocols, and mental health support. Border controls and travel strategies must be managed carefully through testing, quarantine, and monitoring to prevent reintroduction of the virus. Robust surveillance systems, including genomic tracking, remain essential to detect and respond to any resurgence early. Transparent communication is critical to maintain public trust and adherence to guidelines. Additionally, countries should focus on pandemic preparedness by updating response plans, investing in R&D for vaccines and therapeutics, and enhancing global health collaboration. Finally, addressing long-term physical and mental health impacts for survivors will be crucial to promote resilience and recovery.

## 4 Conclusions

We have applied the generalized extreme value distribution to model the monthly maximums of daily new cases across a range of countries including Botswana, Lesotho, Namibia, South Africa, Eswatini, Algeria, Egypt, Libya, Morocco, Sudan, Tunisia, Angola, Cameroon, the Central African Republic, Chad, the Democratic Republic of Congo, Congo, Equatorial Guinea, Gabon, Sao Tome, Benin, Burkina Faso, Cape Verde, Cote d'Ivoire, the Gambia, Ghana, Guinea, Guinea Bissau, Liberia, Mali, Mauritania, Niger, Nigeria, Senegal, Sierra Leone, Togo, Burundi, Comoros, Djibouti, Eritrea, Ethiopia, Kenya, Madagascar, Malawi, Mauritius, Mozambique, Rwanda, Seychelles, Somalia, South Sudan, Tanzania, Uganda, Zambia and Zimbabwe. Within this modeling framework, two of the three parameters of the distribution were allowed to vary either linearly or quadratically concerning the month number to accommodate observed trends.

Sao Tome, Liberia, South Sudan, Eswatini, Morocco, Sudan, Burkina Faso, Ghana, Mali, Mauritania, Senegal, Sierra Leone, Togo, Madagascar, Malawi, Egypt, Libya, Tunisia, Angola, Benin, Cote d'Ivoire, Guinea, Niger, Nigeria, Mozambique and Comoros exhibited negative trends. Mauritius and Uganda exhibited positive trends. South Africa, the Democratic Republic of Congo, Gabon, Tanzania, Zambia, Botswana, Lesotho, Namibia, Algeria, Cameroon, Congo, Burundi, Eritrea, Ethiopia, Kenya, Rwanda, Seychelles, Somalia and Zimbabwe exhibited quadratic type trends.

Estimates of 2.5th, 5th, 50th, 95th, and 97.5th percentiles have been furnished for all fifty four countries up to January 2025. Additionally, asymptotic limits of these percentiles have been provided as the month number approaches infinity. To assess the goodness of fit of the models, probability plots and the Kolmogorov-Smirnov test were employed.

There is notable concern regarding the lack of significant downward trends observed in the monthly maximums of daily new cases for Mauritius and Uganda. Additionally, it is troubling that in Uganda and Congo, the asymptotic limits of the 50th, 95th, and 97.5th percentiles are infinite.

Subsequent research aims to explore the potential spatial dependence among COVID-19 cases across the fifty four countries. Additionally, we seek to examine both spatial and temporal dependencies within COVID-19 cases across the same fifty four nations, consider the effects of vaccination and implemented interventions in specific countries and consider the effect of outliers in some countries.

## Data Availability

The datasets presented in this study can be found in online repositories. The names of the repository/repositories and accession number(s) can be found at: https://ourworldindata.org/covid-cases.
