# Supplementary material for: An extreme value analysis of daily new cases of COVID-19 in Africa
Source: Front Public Health. 2025 Jan 31;13:1546404. doi: 10.3389/fpubh.2025.1546404 (PMC11825754; doi:10.3389/fpubh.2025.1546404)
Supplement: Supplementary file 1 [file Supplementary_file_1.pdf]

Appendix: Tables

Table 1: Summary statistics of the maximum of daily new cases.

Country	Minimum	First quartile	Median	Mean	Third quartile	Maximum	SD	Skewness	Kurtosis
Botswana	0.000	17.000	141.000	2151.623	1820.000	40223.000	16112.061	1.775	4.175
Lesotho	0.000	0.000	17.000	149.962	86.000	1591.000	631.626	1.755	4.137
Namibia	0.000	19.000	104.000	316.415	320.000	3077.000	1202.382	1.735	4.102
South Africa	0.000	12.000	1747.000	5360.396	7210.000	37875.000	14588.141	1.633	3.913
Eswatini	0.000	18.000	49.000	150.679	102.000	1642.000	646.627	1.760	4.147
Algeria	0.000	10.000	73.000	306.925	305.000	2521.000	982.283	1.710	4.057
Egypt	0.000	0.000	179.000	608.415	949.000	5516.000	2143.026	1.671	3.979
Libya	0.000	2.000	35.000	703.000	1007.000	5694.000	2222.748	1.648	3.937
Morocco	0.000	113.000	281.000	1563.906	1776.000	12039.000	4672.582	1.685	4.008
Sudan	0.000	0.000	41.000	170.057	218.000	1284.000	497.531	1.659	3.960
Tunisia	0.000	15.000	435.000	2513.717	2439.000	27340.000	10781.944	1.744	4.118
Angola	0.000	23.000	95.000	314.057	263.000	5611.000	2237.541	1.776	4.177
Cameroon	0.000	12.000	162.000	782.774	884.000	8681.000	3415.525	1.738	4.108
Central African Republic	0.000	0.000	10.000	44.717	32.000	467.000	184.452	1.751	4.131
Chad	0.000	0.000	7.000	35.396	25.000	596.000	238.241	1.775	4.174
Democratic Republic of Congo	0.000	43.000	133.000	206.811	262.000	1518.000	575.439	1.674	3.996
Congo	0.000	1.750	38.000	151.327	274.250	792.000	288.856	1.571	4.023
Equitorial Guinea	0.000	0.000	19.500	74.423	79.500	742.000	271.670	1.974	5.015
Gabon	0.000	0.000	38.000	160.094	212.000	1163.000	449.922	1.642	3.927
Sao Tome	0.000	2.000	8.000	28.226	24.000	319.000	125.682	1.755	4.139
Benin	0.000	0.000	49.000	185.075	125.000	3440.000	1376.981	1.778	4.180
Burkina Faso	0.000	0.000	17.000	85.151	61.000	1005.000	398.461	1.759	4.146
Cape Verde	0.000	7.000	45.000	113.377	125.000	1469.000	578.379	1.756	4.141
Cote d'Ivoire	0.000	8.000	58.000	205.509	198.000	2858.000	1132.046	1.763	4.154
Gambia	0.000	0.000	6.000	41.755	31.000	376.000	148.087	1.735	4.101
Ghana	0.000	18.000	176.000	408.830	716.000	2702.000	1031.251	1.533	3.712
Guinea	0.000	3.000	57.000	89.151	139.000	438.000	164.005	1.398	3.490
Guinea-Bissau	0.000	0.000	13.000	46.019	62.000	305.000	117.394	1.609	3.868
Liberia	0.000	0.000	5.500	20.519	19.250	219.000	80.344	1.991	5.054
Mali	0.000	2.000	25.000	67.792	59.000	1217.000	485.101	1.775	4.176
Mauritania	0.000	5.000	41.000	144.941	97.000	2545.000	945.253	2.027	5.135
Niger	0.000	0.000	9.000	24.189	25.000	301.000	118.650	1.754	4.137
Nigeria	0.000	13.000	159.000	425.736	590.000	4035.000	1567.792	1.702	4.040
Senegal	0.000	4.000	19.000	135.604	118.000	1722.000	682.970	1.759	4.146
Sierra Leone	0.000	0.000	3.000	20.094	18.000	160.000	62.605	1.709	4.053
Togo	0.000	3.000	15.000	67.189	46.000	747.000	295.411	1.758	4.143
Burundi	0.000	14.000	48.000	178.321	160.000	1576.000	615.256	1.731	4.094
Comoros	0.000	0.000	9.000	24.887	20.000	241.000	94.539	1.743	4.116
Djibouti	0.000	0.000	6.000	38.057	26.000	280.000	109.670	1.712	4.058
Eritrea	0.000	0.000	6.000	28.830	25.000	245.000	95.948	1.722	4.078
Ethiopia	0.000	23.000	137.000	658.358	805.000	5185.000	2012.926	1.678	3.996
Kenya	0.000	19.000	171.000	495.887	727.000	3749.000	1443.481	1.639	3.921
Madagascar	0.000	11.000	66.000	279.000	324.000	2984.000	1170.817	1.734	4.100
Malawi	0.000	12.750	30.500	151.500	109.500	1316.000	481.733	1.980	5.028
Mauritius	0.000	21.000	234.000	583.604	824.000	3503.000	1334.056	1.564	3.784
Mozambique	0.000	13.000	62.000	452.811	204.000	6640.000	2656.426	1.773	4.170
Rwanda	0.000	5.000	45.000	239.245	155.000	2773.000	1099.794	1.760	4.147
Seychelles	0.000	1.000	50.000	136.981	184.000	1042.000	401.911	1.656	3.956
Somalia	0.000	0.000	35.000	121.604	136.000	1066.000	415.507	1.709	4.055
South Sudan	0.000	0.000	20.000	55.340	59.000	444.000	172.240	1.700	4.039
Tanzania	0.000	0.000	24.000	563.679	150.000	20210.000	8193.298	1.786	4.195
Uganda	0.000	10.250	76.000	721.000	331.500	20692.000	7753.549	2.036	5.156
Zambia	0.000	35.000	199.000	671.057	720.000	5555.000	2157.562	1.707	4.051
Zimbabwe	0.000	31.000	128.000	451.509	319.000	6181.000	2453.617	1.769	4.164

Table 2: Parameter estimates and standard errors (obtained by simulation) when the constant model was the best fitting model.

Country	Estimates (standard errors)	$-\log L$	AIC	BIC
Central African Republic	$\widehat{a_1} = 2.632$ (0.110), $\widehat{a_2} = 2.773$ (0.192), $\widehat{a_3} = 1.162$ (0.110)	184.092	374.184	379.017
Chad	$\widehat{a_1} = 2.002$ (0.105), $\widehat{a_2} = 2.240$ (0.222), $\widehat{a_3} = 1.173$ (0.106)	173.660	353.321	358.312
Equitorial Guinea	$\widehat{a_1} = 3.314$ (0.133), $\widehat{a_2} = 3.570$ (0.305), $\widehat{a_3} = 0.964$ (0.048)	198.312	402.625	407.291
Cape Verde	$\widehat{a_1} = 3.500$ (0.045), $\widehat{a_2} = 3.706$ (0.328), $\widehat{a_3} = 0.930$ (0.013)	254.643	515.286	520.639
Gambia	$\widehat{a_1} = 2.167$ (0.076), $\widehat{a_2} = 2.519$ (0.053), $\widehat{a_3} = 1.374$ (0.069)	183.614	373.228	378.141
Guinea-Bissau	$\widehat{a_1} = 2.846$ (0.082), $\widehat{a_2} = 2.990$ (0.298), $\widehat{a_3} = 1.188$ (0.106)	196.595	399.191	404.104
Djibouti	$\widehat{a_1} = 2.811$ (0.238), $\widehat{a_2} = 2.879$ (0.012), $\widehat{a_3} = 1.146$ (0.095)	144.989	295.977	299.974

Table 3: Parameter estimates and standard errors (obtained by simulation) when the linear location model was the best fitting model.

Country	Estimates (standard errors)	$-\log L$	AIC	BIC
Sao Tome	$\widehat{a_1} = 1.970$ (0.144), $\widehat{a_2} = 1.858$ (0.004), $\widehat{a_3} = 1.495$ (0.008), $\widehat{b_1} = -1.284$ (0.081)	197.842	403.684	411.084
Liberia	$\widehat{a_1} = 2.068$ (0.137), $\widehat{a_2} = 1.982$ (0.019), $\widehat{a_3} = 1.060$ (0.025), $\widehat{b_1} = -0.474$ (0.005)	144.944	297.889	304.110
South Sudan	$\widehat{a_1} = 3.744$ (0.312), $\widehat{a_2} = 3.009$ (0.002), $\widehat{a_3} = 1.149$ (0.019), $\widehat{b_1} = -2.471$ (0.157)	176.401	360.802	366.907
Uganda	$\widehat{a_1} = 4.236$ (0.378), $\widehat{a_2} = 4.639$ (0.335), $\widehat{a_3} = 1.412$ (0.055), $\widehat{b_1} = 0.515$ (0.033)	279.588	567.176	573.932

Table 4: Parameter estimates and standard errors (obtained by simulation) when the quadratic location model was the best fitting model.

Country	Estimates (standard errors)	$-\log L$	AIC	BIC
South Africa	$\widehat{a_1}$ = 6.418 (0.198), $\widehat{a_2}$ = 6.971 (0.467),<br>$\widehat{a_3}$ = 14.224 (0.474),<br>$\widehat{b_1}$ = 14.224 (0.858), $\widehat{c_1}$ = -39.813 (1.700)	399.492	808.985	817.673
Democratic Republic of Congo	$\widehat{a_1}$ = 4.431 (0.235), $\widehat{a_2}$ = 4.498 (0.415),<br>$\widehat{a_3}$ = 3.717 (0.294),<br>$\widehat{b_1}$ = 3.717 (0.269), $\widehat{c_1}$ = -9.999 (0.132)	296.729	603.458	612.601
Gabon	$\widehat{a_1}$ = 1.298 (0.033), $\widehat{a_2}$ = 2.267 (0.116),<br>$\widehat{a_3}$ = 44.573 (0.919),<br>$\widehat{b_1}$ = 44.573 (0.955), $\widehat{c_1}$ = -158.242 (10.394)	242.408	494.816	503.134
Tanzania	$\widehat{a_1}$ = 3.714 (0.331), $\widehat{a_2}$ = 4.541 (0.250),<br>$\widehat{a_3}$ = 7.281 (0.122),<br>$\widehat{b_1}$ = 7.281 (0.324), $\widehat{c_1}$ = -24.811 (2.198)	203.636	417.272	424.278
Zambia	$\widehat{a_1}$ = 4.836 (0.264), $\widehat{a_2}$ = 5.335 (0.082),<br>$\widehat{a_3}$ = 3.074 (0.015),<br>$\widehat{b_1}$ = 3.074 (0.307), $\widehat{c_1}$ = -7.067 (0.086)	339.909	689.818	698.851

Table 5: Parameter estimates and standard errors (obtained by simulation) when the linear location and linear scale model was the best fitting model.

Country	Estimates (standard errors)	$-\log L$	AIC	BIC
Eswatini	$\widehat{a_1}$ = 4.558 (0.089), $\widehat{a_2}$ = 4.818 (0.337),<br>$\widehat{a_3}$ = 1.020 (0.064),<br>$\widehat{b_1}$ = -4.840 (0.006), $\widehat{b_2}$ = -4.261 (0.026)	285.576	581.152	590.712
Morocco	$\widehat{a_1}$ = 7.360 (0.032), $\widehat{a_2}$ = 7.692 (0.201),<br>$\widehat{a_3}$ = 1.172 (0.072),<br>$\widehat{b_1}$ = -7.986 (0.146), $\widehat{b_2}$ = -7.551 (0.175)	392.916	795.832	805.392
Sudan	$\widehat{a_1}$ = 5.874 (0.374), $\widehat{a_2}$ = 6.314 (0.563),<br>$\widehat{a_3}$ = 1.049 (0.014),<br>$\widehat{b_1}$ = -12.028 (0.487), $\widehat{b_2}$ = -8.254 (0.493)	231.170	472.341	480.529
Burkina Faso	$\widehat{a_1}$ = 4.193 (0.090), $\widehat{a_2}$ = 4.269 (0.391),<br>$\widehat{a_3}$ = 1.342 (0.034),<br>$\widehat{b_1}$ = -6.417 (0.128), $\widehat{b_2}$ = -6.653 (0.074)	207.540	425.080	433.398
Ghana	$\widehat{a_1}$ = 6.716 (0.483), $\widehat{a_2}$ = 6.895 (0.604),<br>$\widehat{a_3}$ = 0.946 (0.000),<br>$\widehat{b_1}$ = -8.895 (0.862), $\widehat{b_2}$ = -8.511 (0.223)	306.928	623.855	632.776
Mali	$\widehat{a_1}$ = 5.447 (0.447), $\widehat{a_2}$ = 5.949 (0.400),<br>$\widehat{a_3}$ = 1.485 (0.111),<br>$\widehat{b_1}$ = -15.681 (1.302), $\widehat{b_2}$ = -12.539 (0.443)	207.851	425.702	434.736
Mauritania	$\widehat{a_1}$ = 4.872 (0.075), $\widehat{a_2}$ = 5.262 (0.503),<br>$\widehat{a_3}$ = 1.113 (0.021),<br>$\widehat{b_1}$ = -8.442 (0.677), $\widehat{b_2}$ = -7.837 (0.255)	257.442	524.883	534.239
Senegal	$\widehat{a_1}$ = 5.466 (0.041), $\widehat{a_2}$ = 5.639 (0.426),<br>$\widehat{a_3}$ = 0.980 (0.024),<br>$\widehat{b_1}$ = -10.815 (0.847), $\widehat{b_2}$ = -10.534 (0.704)	242.785	495.570	504.821
Sierra Leone	$\widehat{a_1}$ = 3.560 (0.165), $\widehat{a_2}$ = 3.210 (0.096),<br>$\widehat{a_3}$ = 1.036 (0.097),<br>$\widehat{b_1}$ = -8.496 (0.708), $\widehat{b_2}$ = -9.636 (0.214)	136.853	283.707	291.624
Togo	$\widehat{a_1}$ = 4.484 (0.404), $\widehat{a_2}$ = 4.829 (0.453),<br>$\widehat{a_3}$ = 1.250 (0.117),<br>$\widehat{b_1}$ = -10.525 (0.429), $\widehat{b_2}$ = -8.883 (0.769)	211.755	433.510	442.653
Madagascar	$\widehat{a_1}$ = 5.636 (0.528), $\widehat{a_2}$ = 5.987 (0.120),<br>$\widehat{a_3}$ = 1.050 (0.031),<br>$\widehat{b_1}$ = -8.200 (0.346), $\widehat{b_2}$ = -7.732 (0.235)	303.430	616.860	626.420
Malawi	$\widehat{a_1}$ = 4.316 (0.415), $\widehat{a_2}$ = 4.541 (0.235),<br>$\widehat{a_3}$ = 1.037 (0.102),<br>$\widehat{b_1}$ = -4.334 (0.009), $\widehat{b_2}$ = -3.707 (0.369)	251.653	513.307	522.228

Table 6: Parameter estimates and standard errors (obtained by simulation) when the quadratic location and linear scale model was the best fitting model.

Country	Estimates (standard errors)	$-\log L$	AIC	BIC
Egypt	$\widehat{a_1}$ = 3.549 (0.126), $\widehat{a_2}$ = 4.950 (0.218), $\widehat{a_3}$ = 2.881 (0.032), $\widehat{b_1}$ = -7.412 (0.098), $\widehat{b_2}$ = 28.024 (1.942), $\widehat{c_1}$ = -93.152 (3.521)	283.022	578.045	588.026
Libya	$\widehat{a_1}$ = 9.988 (0.383), $\widehat{a_2}$ = 12.135 (0.627), $\widehat{a_3}$ = 4.019 (0.166), $\widehat{b_1}$ = -37.571 (1.624), $\widehat{b_2}$ = -22.998 (0.093), $\widehat{c_1}$ = -2.374 (0.049)	296.684	605.368	615.794
Tunisia	$\widehat{a_1}$ = 3.550 (0.236), $\widehat{a_2}$ = 6.135 (0.091), $\widehat{a_3}$ = 5.991 (0.558), $\widehat{b_1}$ = -2.383 (0.060), $\widehat{b_2}$ = 24.636 (1.136), $\widehat{c_1}$ = -57.431 (1.371)	346.990	705.980	716.113
Angola	$\widehat{a_1}$ = 4.238 (0.304), $\widehat{a_2}$ = 4.889 (0.364), $\widehat{a_3}$ = 1.247 (0.079), $\widehat{b_1}$ = -3.518 (0.000), $\widehat{b_2}$ = 7.212 (0.007), $\widehat{c_1}$ = -25.243 (0.775)	301.417	614.834	626.061
Benin	$\widehat{a_1}$ = 6.015 (0.155), $\widehat{a_2}$ = 7.073 (0.017), $\widehat{a_3}$ = 2.619 (0.178), $\widehat{b_1}$ = -16.864 (1.024), $\widehat{b_2}$ = -10.839 (0.453), $\widehat{c_1}$ = -7.297 (0.574)	231.545	475.089	485.070
Cote d'Ivoire	$\widehat{a_1}$ = 5.454 (0.377), $\widehat{a_2}$ = 5.810 (0.353), $\widehat{a_3}$ = 1.141 (0.036), $\widehat{b_1}$ = -9.774 (0.058), $\widehat{b_2}$ = -3.619 (0.121), $\widehat{c_1}$ = -13.815 (0.340)	262.125	536.250	547.221
Guinea	$\widehat{a_1}$ = 4.596 (0.184), $\widehat{a_2}$ = 4.677 (0.060), $\widehat{a_3}$ = 0.508 (0.005), $\widehat{b_1}$ = -4.677 (0.265), $\widehat{b_2}$ = 2.469 (0.062), $\widehat{c_1}$ = -18.745 (0.219)	222.969	457.938	468.071
Niger	$\widehat{a_1}$ = 2.563 (0.078), $\widehat{a_2}$ = 3.326 (0.250), $\widehat{a_3}$ = 0.771 (0.069), $\widehat{b_1}$ = -5.982 (0.572), $\widehat{b_2}$ = 7.040 (0.570), $\widehat{c_1}$ = -30.502 (2.011)	158.804	329.608	339.433
Nigeria	$\widehat{a_1}$ = 5.978 (0.242), $\widehat{a_2}$ = 6.504 (0.153), $\widehat{a_3}$ = 1.085 (0.071), $\widehat{b_1}$ = -7.457 (0.247), $\widehat{b_2}$ = -1.875 (0.070), $\widehat{c_1}$ = -11.604 (0.880)	301.970	615.939	626.507
Mauritius	$\widehat{a_1}$ = 0.227 (0.019), $\widehat{a_2}$ = 1.577 (0.085), $\widehat{a_3}$ = 1.824 (0.061), $\widehat{b_1}$ = 10.359 (0.335), $\widehat{b_2}$ = 28.941 (0.063), $\widehat{c_1}$ = -38.269 (2.132)	337.163	686.325	697.676
Mozambique	$\widehat{a_1}$ = 4.896 (0.387), $\widehat{a_2}$ = 5.493 (0.190), $\widehat{a_3}$ = 1.568 (0.122), $\widehat{b_1}$ = -6.072 (0.427), $\widehat{b_2}$ = -2.635 (0.037), $\widehat{c_1}$ = -7.105 (0.196)	307.210	626.420	637.647

Table 7: Parameter estimates and standard errors (obtained by simulation) when the linear location and quadratic scale model was the best fitting model.

Country	Estimates (standard errors)	$-\log L$	AIC	BIC
Comoros	$\widehat{a_1}$ = 2.997 (0.186), $\widehat{a_2}$ = 1.991 (0.153), $\widehat{a_3}$ = 1.155 (0.108), $\widehat{b_1}$ = 7.173 (0.358), $\widehat{b_2}$ = -31.663 (2.719), $\widehat{c_2}$ = -3.859 (0.103)	147.234	306.468	315.969

Table 8: Parameter estimates and standard errors (obtained by simulation) when the quadratic location and quadratic scale model was the best fitting model.

Country	Estimates (standard errors)	$-\log L$	AIC	BIC
Botswana	$\widehat{a_1}$ = 5.625 (0.233), $\widehat{a_2}$ = 6.040 (0.440),<br>$\widehat{a_3}$ = 1.183 (0.007), $\widehat{b_1}$ = 9.737 (0.834),<br>$\widehat{b_2}$ = -38.593 (2.515), $\widehat{c_1}$ = 6.824 (0.039),<br>$\widehat{c_2}$ = -29.744 (0.225)	373.828	761.656	775.040
Lesotho	$\widehat{a_1}$ = 3.623 (0.329), $\widehat{a_2}$ = 5.251 (0.467),<br>$\widehat{a_3}$ = 5.490 (0.479), $\widehat{b_1}$ = 6.655 (0.038),<br>$\widehat{b_2}$ = -81.206 (0.398), $\widehat{c_1}$ = 1.227 (0.006),<br>$\widehat{c_2}$ = -14.929 (1.104)	197.385	408.771	419.246
Namibia	$\widehat{a_1}$ = 2.487 (0.232), $\widehat{a_2}$ = 2.804 (0.118),<br>$\widehat{a_3}$ = 0.816 (0.072), $\widehat{b_1}$ = 27.600 (0.419),<br>$\widehat{b_2}$ = -66.263 (3.701), $\widehat{c_1}$ = 27.007 (1.363),<br>$\widehat{c_2}$ = -61.476 (5.432)	304.942	623.884	637.127
Algeria	$\widehat{a_1}$ = 5.006 (0.021), $\widehat{a_2}$ = 5.103 (0.064),<br>$\widehat{a_3}$ = 0.662 (0.049), $\widehat{b_1}$ = 8.469 (0.356),<br>$\widehat{b_2}$ = -39.818 (1.985), $\widehat{c_1}$ = 6.685 (0.314),<br>$\widehat{c_2}$ = -32.781 (0.914)	290.154	594.308	607.551
Cameroon	$\widehat{a_1}$ = 4.143 (0.408), $\widehat{a_2}$ = 3.908 (0.019),<br>$\widehat{a_3}$ = 0.806 (0.044), $\widehat{b_1}$ = 36.610 (3.538),<br>$\widehat{b_2}$ = -103.824 (2.872), $\widehat{c_1}$ = 35.662 (2.488),<br>$\widehat{c_2}$ = -106.257 (4.476)	311.250	636.499	648.989
Congo	$\widehat{a_1}$ = 0.003 (0.000), $\widehat{a_2}$ = 1.521 (0.063),<br>$\widehat{a_3}$ = 5.042 (0.183), $\widehat{b_1}$ = 55.188 (2.910),<br>$\widehat{b_2}$ = -204.092 (16.215), $\widehat{c_1}$ = -0.015 (0.001),<br>$\widehat{c_2}$ = 0.018 (0.000)	234.581	483.161	495.490
Burundi	$\widehat{a_1}$ = 1.087 (0.083), $\widehat{a_2}$ = 0.582 (0.035),<br>$\widehat{a_3}$ = 0.800 (0.027), $\widehat{b_1}$ = 34.868 (0.589),<br>$\widehat{b_2}$ = -69.388 (3.525), $\widehat{c_1}$ = 31.962 (0.534),<br>$\widehat{c_2}$ = -66.338 (1.038)	267.292	548.584	561.535
Eritrea	$\widehat{a_1}$ = 1.867 (0.088), $\widehat{a_2}$ = 1.358 (0.131),<br>$\widehat{a_3}$ = 0.351 (0.031), $\widehat{b_1}$ = 34.009 (2.830),<br>$\widehat{b_2}$ = -117.937 (7.447), $\widehat{c_1}$ = 28.983 (0.141),<br>$\widehat{c_2}$ = -105.023 (4.982)	140.947	295.893	306.154
Ethiopia	$\widehat{a_1}$ = 5.575 (0.279), $\widehat{a_2}$ = 5.869 (0.118),<br>$\widehat{a_3}$ = 0.734 (0.010), $\widehat{b_1}$ = 9.510 (0.358),<br>$\widehat{b_2}$ = -41.262 (1.888), $\widehat{c_1}$ = 12.062 (0.848),<br>$\widehat{c_2}$ = -45.747 (4.028)	330.912	675.824	689.208
Kenya	$\widehat{a_1}$ = 5.250 (0.516), $\widehat{a_2}$ = 5.465 (0.409),<br>$\widehat{a_3}$ = 0.716 (0.043), $\widehat{b_1}$ = 7.803 (0.744),<br>$\widehat{b_2}$ = -33.589 (1.999), $\widehat{c_1}$ = 9.038 (0.706),<br>$\widehat{c_2}$ = -35.922 (3.590)	316.168	646.336	659.137
Rwanda	$\widehat{a_1}$ = 2.495 (0.020), $\widehat{a_2}$ = 1.375 (0.099),<br>$\widehat{a_3}$ = 1.017 (0.041), $\widehat{b_1}$ = 38.426 (3.265),<br>$\widehat{b_2}$ = -97.337 (5.310), $\widehat{c_1}$ = 27.460 (1.366),<br>$\widehat{c_2}$ = -73.682 (7.156)	235.190	484.379	496.201
Seychelles	$\widehat{a_1}$ = -0.445 (0.021), $\widehat{a_2}$ = -0.307 (0.007),<br>$\widehat{a_3}$ = 0.770 (0.075), $\widehat{b_1}$ = 48.253 (0.916),<br>$\widehat{b_2}$ = -106.832 (2.032), $\widehat{c_1}$ = 50.260 (2.918),<br>$\widehat{c_2}$ = -110.087 (10.319)	227.792	469.584	481.406
Somalia	$\widehat{a_1}$ = 2.410 (0.038), $\widehat{a_2}$ = 2.543 (0.098),<br>$\widehat{a_3}$ = 0.305 (0.012), $\widehat{b_1}$ = 32.672 (1.984),<br>$\widehat{b_2}$ = -101.608 (3.161), $\widehat{c_1}$ = 27.933 (2.550),<br>$\widehat{c_2}$ = -82.492 (4.612)	211.941	437.882	449.159
Zimbabwe	$\widehat{a_1}$ = 3.495 (0.177), $\widehat{a_2}$ = 3.679 (0.250),<br>$\widehat{a_3}$ = 0.692 (0.025), $\widehat{b_1}$ = 20.277 (1.652),<br>$\widehat{b_2}$ = -52.076 (4.488), $\widehat{c_1}$ = 21.603 (1.952),<br>$\widehat{c_2}$ = -53.597 (0.900)	318.297	650.595	663.979

Table 9: Asymptotic limits of q percent quantile.

Country	$q = 97.5$	$q = 95$	$q = 50$	$q = 5$	$q = 2.5$
Central African Republic	647	283	11	0	0
Chad	414	180	6	0	0
Equitorial Guinea	855	426	18	0	0
Cape Verde	1110	569	37	0	0
Gambia	887	334	7	0	0
Guinea-Bissau	884	380	14	0	0
Djibouti	500	220	6	0	0
Sao Tome	867	298	1	0	0
Liberia	213	97	0	0	0
South Sudan	701	299	0	0	0
Uganda	∞	∞	∞	0	0
South Africa	866643	184953	3	0	0
Democratic Republic of Congo	1333	772	14	0	0
Gabon	11477	2141	0	0	0
Tanzania	62929	11389	0	0	0
Zambia	12640	5173	26	0	0
Eswatini	0	0	0	0	0
Morocco	0	0	0	0	0
Sudan	0	0	0	0	0
Burkina Faso	0	0	0	0	0
Ghana	0	0	0	0	0
Mali	0	0	0	0	0
Mauritania	0	0	0	0	0
Senegal	0	0	0	0	0
Sierra Leone	0	0	0	0	0
Togo	0	0	0	0	0
Madagascar	0	0	0	0	0
Malawi	0	0	0	0	0
Egypt	739143	95271	0	0	0
Libya	32219555149	1860126084	409	0	0
Tunisia	49939114156	707980342	0	0	0
Angola	8780	3574	32	0	0
Benin	2554092	396675	0	0	0
Cote d'Ivoire	14681	6396	51	0	0
Guinea	929	586	0	0	0
Niger	412	224	0	0	0
Nigeria	23934	10781	27	0	0
Mauritius	2074	569	2	0	0
Mozambique	39514	12937	60	0	0
Comoros	0	0	0	0	0
Botswana	0	0	0	0	0
Lesotho	0	0	0	0	0
Namibia	0	0	0	0	0
Algeria	0	0	0	0	0
Cameroon	0	0	0	0	0
Congo	∞	∞	∞	0	0
Burundi	0	0	0	0	0
Eritrea	0	0	0	0	0
Ethiopia	0	0	0	0	0
Kenya	0	0	0	0	0
Rwanda	0	0	0	0	0
Seychelles	0	0	0	0	0
Somalia	0	0	0	0	0
Zimbabwe	0	0	0	0	0
